# Association of blood cadmium, lead, and mercury with anxiety: a cross-sectional study from NHANES 2007–2012

**DOI:** 10.3389/fpubh.2024.1402715

**Published:** 2024-08-12

**Authors:** Long Bai, Zongliang Wen, Yan Zhu, Hamdi Abdirizak Jama, Joyce D. Sawmadal, Jialin Chen

**Affiliations:** ^1^School of Public Health, Xuzhou Medical University, Xuzhou, China; ^2^School of Management, Xuzhou Medical University, Xuzhou, China; ^3^Affiliated Hospital of Xuzhou Medical University, Xuzhou, China; ^4^School of Medical Information and Engineering, Xuzhou Medical University, Xuzhou, China

**Keywords:** cadmium, lead, mercury, anxiety, restricted cubic spline, NHANES

## Abstract

**Objectives:**

The purpose of this paper is to explore the relationship between blood levels of cadmium, lead, and mercury and anxiety in American adults.

**Methods:**

Blood metals and self-reported anxiety days were extracted from laboratory data and questionnaire data, respectively, using NHANES data from 2007–2012. Weighted logistic regression was used to assess the relationship between cadmium, lead and mercury with anxiety. Restricted cubic spline was used to visualize the non-linear relationship between metal concentrations and anxiety. Weighted quantile sum (WQS) regression was used to investigate the effect of combined exposure to the three metals on anxiety.

**Results:**

The prevalence of anxiety in adults was 26.0%. After adjusting for potential confounding variables, cadmium levels in the highest quartile (Q4) were associated with a higher risk of anxiety compared to the lowest quartile (Q1) (OR = 1.279, 95% CI: 1.113–1.471, *p* < 0.01). Restricted cubic spline analysis indicated a positive association between blood cadmium levels and anxiety. Furthermore, co-exposure to multiple heavy metals was positively associated with anxiety risk (WQS positive: OR = 1.068, 95% CI: 1.016–1.160, *p* < 0.05), with cadmium contributing the most to the overall mixture effect. Compared to the Light RPA, the Vigorous/Moderate RPA group had a relatively low risk of anxiety after cadmium exposure.

**Conclusion:**

High levels of blood cadmium are positively associated with the development of anxiety disorders, which needs to be further verified in future studies.

## 1 Introduction

Concerns about mental health-related issues, particularly anxiety disorders, have been increasingly expressed by the public. Anxiety is one of the manifestations of a wide range of mental disorders, characterized by acute, overwhelming, and persistent worry and fear that can peak within minutes (1). Anxiety is a common disorder that can have a detrimental effect on quality of life (QOL), especially when untreated (2). A study on the global burden of anxiety disorders from 1990 to 2019 showed a 50% increase in the absolute number of anxiety disorders compared to 1990 (3). Excessive anxiety constitutes the most common psychiatric complaint (4). The causes of anxiety are complex and varied, including life stress, illness, and exposure to environmental pollutants (5, 6). The relationship between environmental chemicals, such as heavy metals, and the etiology of mental disorders has garnered widespread attention (7).

Heavy metals, defined as metals with a density >5 g/cm3 (e.g., mercury [Hg], lead [Pb], and cadmium [Cd]), are non-essential and highly toxic to humans (8). Metals are present in almost all environmental media in everyday life, and people are often exposed to many types of metals at once. Cadmium is a major contaminant in agricultural soils worldwide and its toxicity and persistence in the environment has become a matter of concern (9, 10). Lead is widely used in the weapons, paint and battery industries, and was previously widely used in plumbing and food packaging, and is released from sources such as batteries (11). Mercury is typically found in elemental form and as methylmercury, the latter being highly toxic (12). Prolonged exposure to these environmental heavy metals, even if they are essential elements required by the human body, can have adverse effects on human health when certain threshold levels are exceeded (13). It has been shown that heavy metal pollution can negatively impact mental health (14). An epidemiological study has linked living in areas with high concentrations of heavy metals and metalloids in the soil to an increased likelihood of developing mental disorders. In this study, compared to the lowest metal concentration level quartile, the odds ratios (OR) for the second, third and fourth quartiles for lead were 1.29, 1.37 and 1.51, respectively; and for cadmium, the OR for the fourth quartile was 1.84 (15). Heavy metals are known to be severely neurotoxic, causing brain damage by interfering with the release of neurotransmitters and neurotrophic proteins, generating neuroinflammation and oxidative stress, and leading to necrosis and apoptosis of neurons and glial cells, which may be a potential mechanism for psychiatric disorders (16–18), and which are often dependent on the relevance of the dose and exposure window.

Many reports have demonstrated the relationship between heavy metal exposure and mental disorders. It has been noted that joint exposure to metals is associated with elevated anxiety symptoms (OR_WQS_ series = 1.56, 95% CI: 1.24, 1.96); Cd (61.8%), Cr (14.7%), and Cs (12.7%) contributed most to the mixed effect (19). A study based on data from the National Health and Nutrition Examination Survey (NHANES) showed a positive correlation between higher levels of cadmium and depression (20). Lead exposure is also positively associated with the development of anxiety and depression (21). Despite evidence that heavy metal exposure increases the risk of psychiatric disorders, only a few studies have shown that heavy metal exposure may increase anxiety (7, 19, 22). In a study examining the effect of whole blood lead and cadmium levels on the prevalence of anxiety and depressive symptoms in postmenopausal women, a relationship was found between lead levels and the severity of anxiety states. Those without anxiety had the highest whole blood lead concentration (22.84 ± 9.79 μg/L), while those with anxiety had the lowest whole blood lead concentration (17.20 ± 7.52 μg/L), a difference that was statistically significant (22). However, some meta-analyses have found no evidence of a correlation between lead exposure (assessed as blood lead levels) and anxiety (23). A recent study exploring the relationship between heavy metals and anxiety found a significant association with urinary metals (7). However, the relationship between blood heavy metal levels and anxiety remains to be fully explored.

Given these conflicting findings, we aimed to investigate whether blood concentrations of three heavy metals (lead, mercury, and cadmium) are associated with anxiety in the American population. We examined cross-sectional associations between anxiety and blood concentrations of these heavy metals among participants in the National Health and Nutrition Examination Survey (NHANES), a randomly selected, non-institutionalized sample of Americans.

## 2 Method

### 2.1 Study population

**Figure 1:**
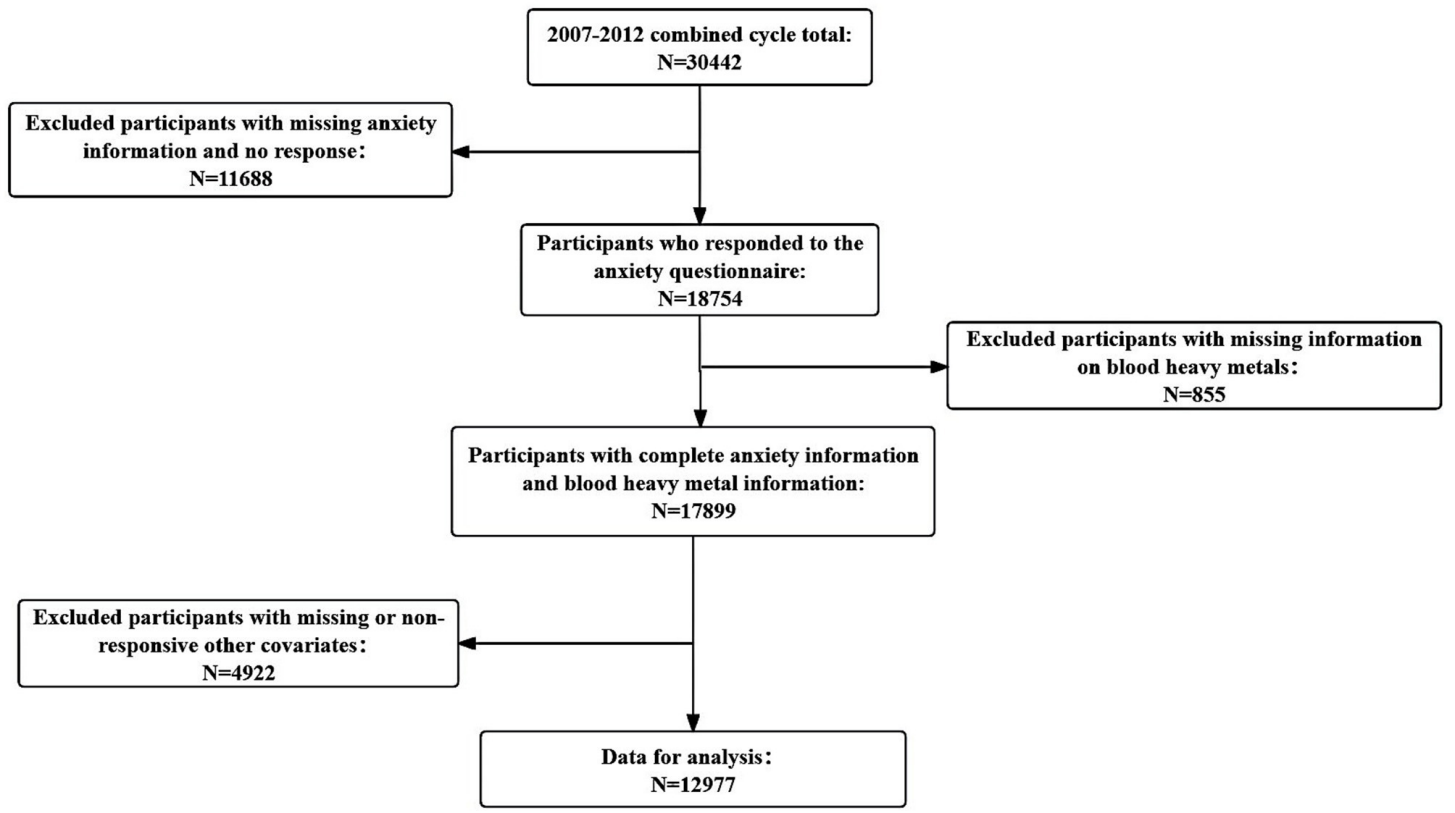
Flowchart for selecting study participants.

The National Health and Nutrition Examination Survey (NHANES) is a series of cross-sectional health and nutrition surveys conducted by the National Centre for Health Statistics. NHANES employs a stratified multistage sampling design. The survey has received approval from the Ethical Review Board of the National Center for Disease Control and Prevention (NCDC), and informed consent was obtained from all participants. Detailed information about the NHANES procedures is available on the official website (https://www.cdc.gov/nchs/nhanes/index.htm). For this study, we used data from the NHANES 2007–2012 survey cycles. We excluded participants younger than 20 years old, those without information on anxiety status, and those with unreliable data. Additionally, individuals with missing information on blood cadmium, lead, and mercury levels were excluded. The specific procedure is illustrated in Figure 1.

### 2.2 Definition of anxiety

During the personal interview, anxiety status was assessed by the following question, “During the past 30 days, about how many days did you feel worried, nervous, or anxious?” This assessment is based on the 14-item Healthy Days measure developed by the CDC, which is part of the Health-Related Quality of Life (HRQoL) assessment. The Reliability of HRQoL monitoring questions ranges from moderate to excellent (24). Anxiety states were categorized as follows: “no” (feeling anxious 0 to 6 days per month) and “yes” (feeling anxious 7–30 days per month) (25).

### 2.3 Measurement of blood metals

Whole blood specimens were processed, stored, and shipped to the Division of Laboratory Sciences, National Center for Environmental Health, and Centers for Disease Control and Prevention for analysis. Detailed instructions on specimen collection and processing can be found in the NHANES Laboratory/Medical Technologists Procedures Manual (LPM). Whole blood lead (Pb), cadmium (Cd), and total mercury (THg) concentrations are determined using inductively coupled plasma mass spectrometry. This multi-element analytical technique is based on quadrupole ICP-MS technology. Produced by ion detection signal is processed into digital information, used to indicate the intensity of the ion, then the concentration of indicator elements. For values below the detection limit, we followed NHANES analytical guidelines by dividing the detection limit by the square root of two.

### 2.4 Covariates

The following variables were included in the statistical analyses: gender (male/female), age (20–39, 40–59, ≥ 60), race (Mexican American, Other Hispanic, non-Hispanic white, non-Hispanic black, or other race), marital status (not living alone/living alone), and education (< high school, high school, or >high school), and poverty income ratio (< 1.50, 1.50–3.49, ≥3.50). Other confounding variables included smoking status (defined as having smoked at least 100 cigarettes in a lifetime) and drinking status (defined as having had at least 12 drinks in a year). Hypertension and diabetes were derived from self-reported physician diagnoses (yes/no). Body mass index was obtained from examination data (< 25.0,25.0–29.99, ≥30.0). Physical activity was classified as light RPA and vigorous/moderate RPA.

### 2.5 Statistical analyses

Considering the complex sampling of NHANES, appropriate weights were used in the statistical analyses, with a sample weight of 1/3 ^*^ WTMEC2YR for the years 2007–2012, the formula for this weight can be found on this website (https://wwwn.cdc.gov/nchs/nhanes/tutorials/Weighting.aspx). Covariates were transformed into categorical variables and expressed as observations and weighted percentages. *P*-values were tested using the weighted chi-square test. Logistic regression was used to examine the association of cadmium, lead and mercury with anxiety. In the crude model, no variables were adjusted; model 1 was adjusted for gender, age, and race; model 2 was adjusted for gender, age, race, education, marital status, BMI, poverty income ratio, smoking status, drinking status, diabetes, hypertension, and physical activity. A restricted cubic spline was used to explore the non-linear relationship between heavy metals and anxiety. Weighted quantile sum (WQS) regression was employed to assess the combined effect of heavy metal exposure on anxiety. The data was divided into 40% training dataset and 60% validation dataset and the positive and negative effects of WQS index on anxiety risk were analyzed by WQS regression. Subgroup analyses based on different characteristic differences (e.g., gender and age) were conducted to assess the effects of heavy metals on anxiety across different demographics. Data were analyzed using SPSS 27.0, Empowerstats (http://www.empowerstats.com), and R version 4.2.2. The R package “gWQS” was used for WQS. A two-sided p-value < 0.05 was considered statistically significant.

## 3 Results

### 3.1 Basic characteristics of participants

Table 1 summarizes the general characteristics of the study participants, including 12,977 individuals, with 6,449 (48.8%) males and 6,528 (51.2%) females. All participants were adults aged ≥20 years: 4,402 (36.8%) were aged 20–39 years, 4,250 (38.6%) were aged 40–59 years, and 4,325 (24.5%) were aged ≥60 years. Among the participants, 4,586 (30.6%) had hypertension, and 1,605 (8.8%) had diabetes. Anxiety was present in 3,360 (26.0%) individuals, of whom 1,354 (41.1%) were male and 2,006 (58.9%) were female. In the anxious population, 1,210 (38.8%) were aged 20–39 years, 1,326 (43.8%) were aged 40–59 years, and 824 (17.4%) were aged ≥60 years. Additionally, 1,503 (40.0%) were living alone, 1,976 (52.1%) engaged in light recreational physical activity (RPA), and 1,384 (47.9%) engaged in vigorous/moderate RPA. All characteristics, except for alcohol drinking status, diabetes, and hypertension, showed statistically significant differences between the anxious and non-anxious groups.

**Table 1:** Characteristics of participants by anxiety status, NHANES 2007–2012, weighted.

	**Total**	**No**	**Yes**	***P* value**
N	12,977 (100%)	9,617 (74.0%)	3,360 (26.0%)	
**Gender, %**				< 0.001
Male	6,449 (48.8)	5,095 (51.5)	1,354 (41.1)	
Female	6,528 (51.2)	4,522 (48.5)	2,006 (58.9)	
**Age, years, %**				< 0.001
20–39	4,402 (36.8)	3,192 (36.2)	1,210 (38.8)	
40–59	4,250 (38.6)	2,924 (36.8)	1,326 (43.8)	
≥60	4,325 (24.5)	3,501 (27.1)	824 (17.4)	
**Race, %**				0.018
Mexican American	1,925 (7.7)	1,436 (7.9)	489 (7.2)	
Other Hispanic	1,289 (5.1)	904 (4.8)	385 (5.9)	
Non-Hispanic White	6,123 (70.7)	4,460 (70.5)	1,663 (71.5)	
Non-Hispanic Black	2,639 (10.3)	2,011 (10.4)	628 (10.1)	
Other Races	1,001 (6.2)	806 (6.5)	195 (5.3)	
**Education, %**				< 0.001
< High school	3,362 (17.2)	2,384 (16.1)	978 (20.5)	
High school	2,978 (22.5)	2,264 (22.8)	714 (21.4)	
>High school	6,637 (60.3)	4,969 (61.1)	1,668 (58.1)	
**Marital status, %**				< 0.001
Not living alone	7,711 (63.5)	5,854 (64.7)	1,857 (60.0)	
Living alone	5,266 (36.5)	3,763 (35.3)	1,503 (40.0)	
**Smoking status, %**				< 0.001
Smoker	5,985 (45.4)	4,256 (43.4)	1,729 (51.1)	
Never smoker	6,992 (54.6)	5,361 (56.6)	1,631 (48.9)	
**Drinking status, %**				0.060
Yes	9,493 (78.2)	6,972 (77.7)	2,521 (79.8)	
No	3,484 (21.8)	2,645 (22.3)	839 (20.2)	
**Diabetes, %**				0.373
Yes	1,605 (8.8)	1,146 (8.6)	459 (9.3)	
No	11,372 (91.2)	8,471 (91.4)	2,901 (90.7)	
**Hypertension, %**				0.128
Yes	4,586 (30.6)	3,337 (30.1)	1,249 (32.0)	
No	8,391 (69.4)	6,280 (69.9)	2,111 (68.0)	
**Body mass index, %**				0.009
< 25.0	3,805 (30.9)	2,835 (30.7)	970 (31.5)	
25.0–29.99	4,353 (33.9)	3,350 (34.8)	1,003 (31.5)	
≥30	4,819 (35.2)	3,432 (34.5)	1,387 (37.0)	
**Poverty income ratio, %**				< 0.001
< 1.50	4,956 (25.9)	3,406 (23.5)	1,550 (32.8)	
1.50–3.49	4,028 (30.5)	3,041 (30.5)	987 (30.7)	
≥3.50	3,993 (43.5)	3,170 (46.0)	823 (36.6)	
**Physical activity, %**				< 0.001
Light RPA	6,832 (45.6)	4,856 (43.4)	1,976 (52.1)	
Vigorous/Moderate RPA	6,145 (54.4)	4,761 (56.6)	1,384 (47.9)	

For categorical variables: (N-observe) survey-weighted percentage, P-value was by survey-weighted Chi-square test; RPA: Recreational Physical Activity.

### 3.2 Relationship between blood metals and anxiety

Table 2 shows the relationship between blood levels of cadmium, lead and mercury with anxiety, categorizing participants according to the interquartile range (IQR) of heavy metal concentrations (Q1: 0–25%; Q2: >25%-50%; Q3: >50%-75%; and Q4: >75%-100%), as shown in Supplementary Table S1. Three logistic regression models were developed, and in the crude model, blood cadmium was positively associated with the risk of anxiety in the Q4 group (OR =1.631, 95% CI: 1.458–1.825, *P* < 0.001), while blood lead in the Q4 group and blood mercury in the Q4 group were negatively associated with the risk of anxiety. After adjusting for gender, age, and race in model 1, the association between blood cadmium and anxiety remained strong (OR = 1.691, 95%CI: 1.501–1.904, *p* < 0.001), and Q2 (OR = 1.172, 95%CI: 1.014–1.356, *p* < 0.05) and Q4 (OR = 1.223, 95%CI: 1.047–1.428, *p* < 0.05) of blood lead were associated with the risk of anxiety. An association between Q4 and anxiety remained for blood mercury (OR = 0.812, 95% CI: 0.700–0.942, *p* < 0.01). In Model 2, after adjusting for gender, age, race, education, marital status, smoking status, drinking status, hypertension, diabetes, body mass index, poverty income ratio, and physical activity, the association between blood cadmium and anxiety persisted (OR = 1.279, 95% CI: 1.113–1.471, *p* < 0.01), while the associations with blood lead and mercury were not statistically significant.

**Table 2:** Relationship between blood metals and anxiety, 2007–2012, weighted.

	**Crude**	**Model 1**	**Model 2**
**Cadmium**			
Q1	Ref	Ref	Ref
Q2	1.051 (0.933, 1.183)	1.063 (0.941, 1.200)	1.047 (0.917, 1.196)
Q3	0.975 (0.853, 1.114)	1.034 (0.898, 1.190)	0.939 (0.820, 1.075)
Q4	1.631 (1.458, 1.825)^***^	1.691 (1.501, 1.904)^***^	1.279 (1.113, 1.471)^**^
*P* for trend	< 0.001	< 0.001	< 0.001
**Lead**			
Q1	Ref	Ref	Ref
Q2	1.029 (0.899, 1.176)	1.172 (1.014, 1.356)^*^	1.101 (0.958, 1.267)
Q3	0.861 (0.737, 1.007)	1.105 (0.930, 1.312)	0.983 (0.825, 1.172)
Q4	0.861 (0.763, 0.972)^*^	1.223 (1.047, 1.428)^*^	1.020 (0.885, 1.175)
*P* for trend	0.005	0.040	0.797
**Mercury**			
Q1	Ref	Ref	Ref
Q2	0.861 (0.741, 1.001)	0.878 (0.754, 1.022)	0.945 (0.813, 1.099)
Q3	0.887 (0.770, 1.022)	0.891 (0.771, 1.030)	1.014 (0.878, 1.172)
Q4	0.779 (0.673, 0.900)^**^	0.812 (0.700, 0.942)^**^	1.011 (0.866, 1.180)
*P* for trend	0.004	0.023	0.670

^*^p < 0.05; ^**^p < 0.01; ^***^p < 0.001.

OR, Odds Ratio; Q, quartile.

Crude: unadjusted.

Model 1: adjusted for gender, age, race.

Model 2: adjusted for gender, age, race, education, marital status, smoking status, drinking status, body mass index, poverty income ratio, hypertension, diabetes, and physical activity.

**Figure 2:**
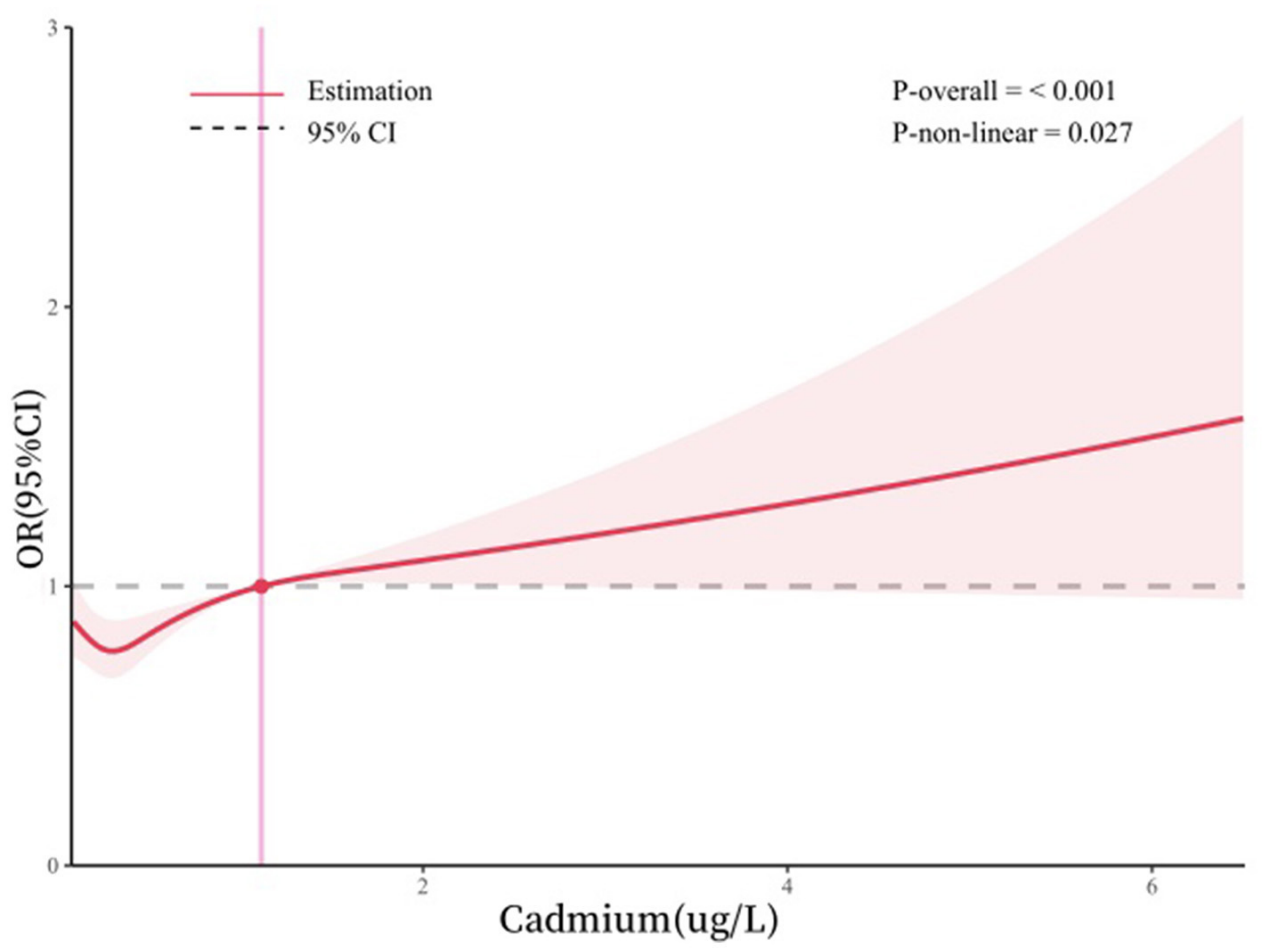
Dose-response relationship between blood cadmium and anxiety. Adjusted for gender, age, race, education, marital status, smoking status, drinking status, body mass index, poverty income ratio, hypertension, diabetes, and physical activity.

Figure 2 illustrates the non-linear relationship between blood cadmium and anxiety derived from the restricted cubic spline model analysis, adjusted for gender, age, race, education, marital status, smoking status, drinking status, hypertension, diabetes, body mass index, poverty income ratio, and physical activity. The analysis showed that high levels of cadmium in the blood were positively associated with anxiety risk.

We also constructed bimetallic and polymetallic models to assess the effects of combined metal exposures, such as cadmium. The results supported our previous findings. After adjusting for covariates in the polymetallic model, blood cadmium remained positively associated with anxiety risk in the Q4 group (Supplementary Table S2).

### 3.3 Effects of cadmium on anxiety under different physical activities

Weighted logistic regression results in Table 3 indicate that vigorous/moderate RPA reduces the risk of anxiety following cadmium exposure compared to light RPA. After adjusting for other variables, the OR and 95% CI for the risk of anxiety after cadmium exposure for vigorous/moderate RPA was 1.132 (1.006–1.274, *p* < 0.05).

**Table 3:** Relationship between blood cadmium and anxiety at different levels of physical activity, weighted.

	**Crude**	**Model 1**	**Model 2**
Light RPA	1.380 (1.196,1.592)^***^	1.361 (1.164,1.590)^***^	1.174 (1.030,1.337)^*^
Vigorous/ Moderate RPA	1.245 (1.120,1.384)^***^	1.237 (1.111,1.377)^***^	1.132 (1.006,1.274)^*^

^*^p < 0.05; ^***^p < 0.001.

RPA, Recreational Physical Activity.

Cadmium is brought in as a continuous variable.

Crude: unadjusted.

Model 1: adjusted for gender, age, race.

Model 2: adjusted for gender, age, race, education, marital status, smoking status, drinking status, body mass index, poverty income ratio, hypertension, and diabetes.

### 3.4 The combined effect of the three metals: weighted quantile sum regression (WQS)

**Figure 3:**
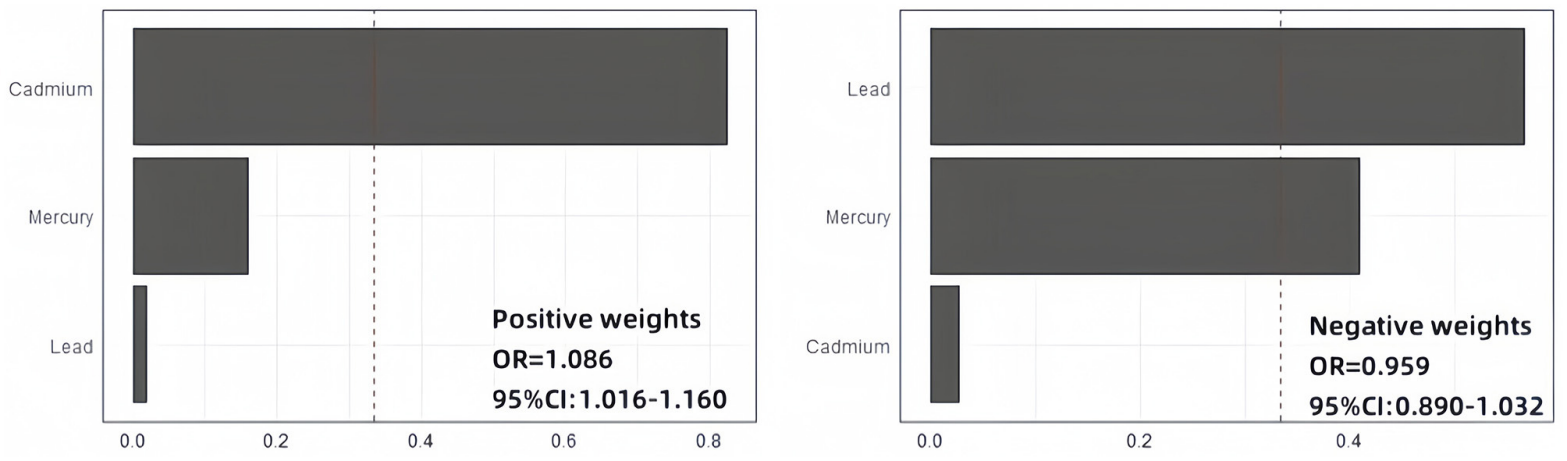
WQS regression results. Adjusted for gender, age, race, education, marital status, smoking status, drinking status, body mass index, poverty income ratio, hypertension, diabetes, and physical activity.

As shown in Figure 3, the weighted quantile sum (WQS) regression model indicated that each one-unit increase in the WQS index for the positive effect of all metals was associated with an 8.6% increase in the risk of anxiety (95% CI: 1.016–1.160). Conversely, for the negative effect of all metals, the overall OR for anxiety was 0.959 (0.890, 1.032). Additionally, the weights of blood heavy metals estimated by the WQS model are presented in Supplementary Table S3. Cadmium had the highest estimated weight for anxiety risk (weight = 0.823), followed by mercury (weight = 0.158). We did not find any significant negative correlation between heavy metal mixtures and anxiety risk.

### 3.5 Subgroup analysis

**Figure 4:**
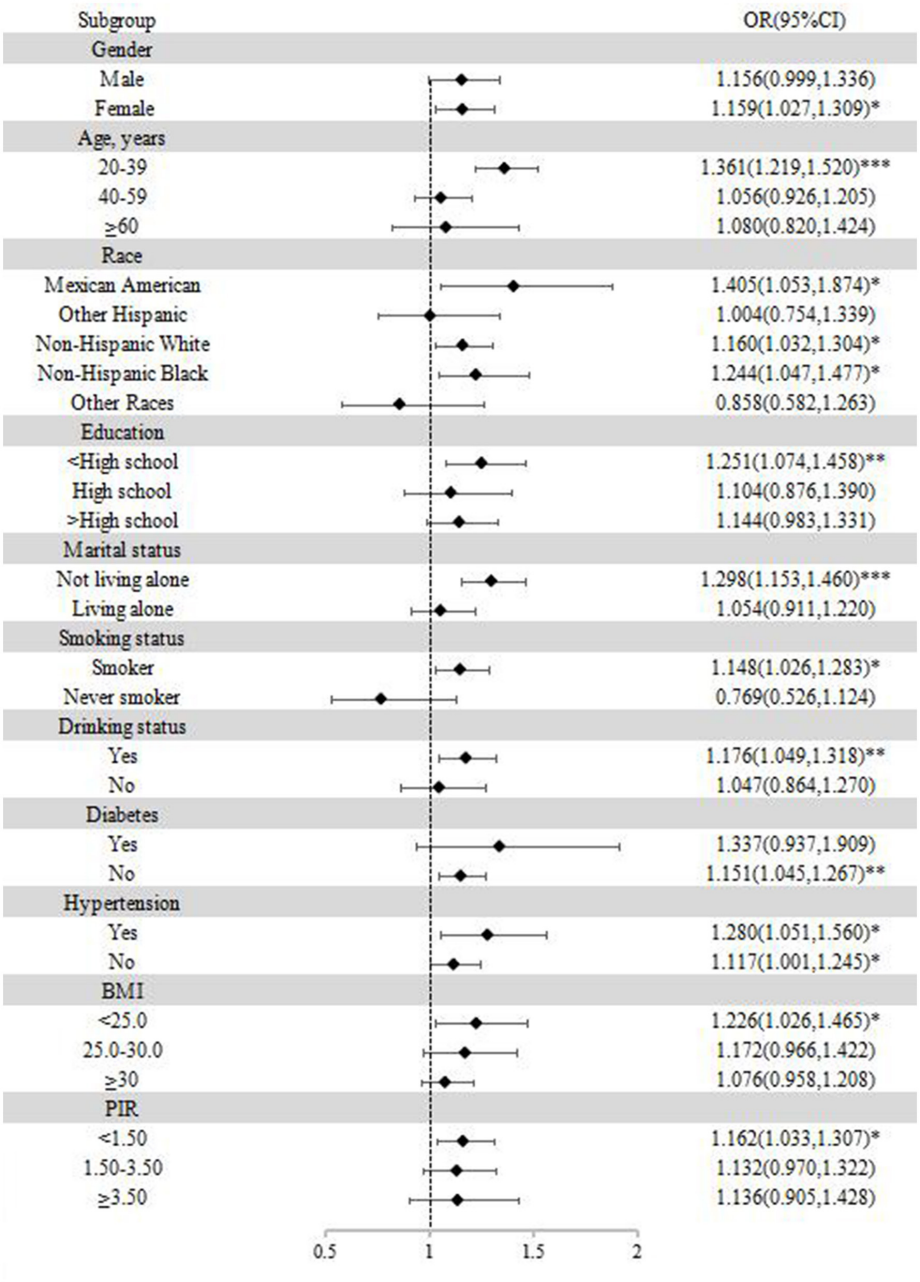
Subgroup analysis of the association between blood cadmium and anxiety. *p < 0.05; **p < 0.01; ***p < 0.001.

In the subgroup analyses, stratification was based on gender, age, race, education, marital status, smoking status, drinking status, body mass index, poverty income ratio, hypertension, and diabetes. As shown in Figure 4, participants who were female, 20–39 years old, under high-school education, not living alone, smoking, drinking alcohol, BMI < 25.0 kg/m^2^, and PIR < 1.50 were more likely to suffer from anxiety.

## 4 Discussion

Heavy metal exposure poses a significant risk to human health. We investigated the relationship between cadmium, lead, and mercury and anxiety using NHANES data from 2007 to 2012, revealing a positive association between blood cadmium levels and anxiety. When stratified by physical activity and adjusted for other variables, the risk of cadmium exposure was lower in the Vigorous/Moderate RPA group compared to the light RPA group (OR: 1.132 < 1.174).

Although the neurotoxicity of cadmium is well known (26), limited studies have explored the association between cadmium exposure and anxiety risk. A recent study found a positive correlation between cadmium levels in urine and anxiety risk (7). Joint exposure to metals was associated with elevated anxiety symptoms, with Cd (61.8%), contributing the most to the mixed effect (19). In another experiment, control rats received intraperitoneal injections of 0.9% NaCl, while test rats were injected with CdCl_2_ dissolved in physiological solution at doses of 1 mg/kg, 2 mg/kg, and 3 mg/kg, and their behavioral activities were observed. The results showed that acute cadmium administration dose-dependently increased anxiety in rats (27). Contrarily, previous studies have suggested that cadmium is not related to anxiety. A systematic evaluation of observational studies found no association between blood cadmium levels and anxiety (23). In our study, blood lead levels were not associated with anxiety after controlling for all covariates. In a study of the association of metal ions (containing lead) in cerebrospinal fluid with anxiety, depression, and insomnia in smokers, no association between lead and anxiety was found (28). A study on the relationship between blood lead exposure and mental health in pregnant women also found no association between low levels of lead exposure and psychological symptoms (29). However, some studies have shown a positive correlation between lead exposure and the onset of anxiety. Intermittent lead exposure can lead to adverse health effects, including anxiety (30). Mercury is a toxic metal that can cause health problems with prolonged exposure. Studies have shown that metalworkers regularly exposed to mercury are at risk of developing anxiety (31). In a study of mercury exposure in the Terra do Meio region of the Amazon, a high prevalence of symptoms associated with mercury poisoning was observed, with anxiety being one of the symptoms (32). Our study found no association between blood mercury levels and anxiety. Some studies have found no relationship between mercury levels in the body and mental disorders (33). A study on the relationship between exposure to environmental pollutants and behavioral indicators in Inuit preschool children in the Québec Arctic region found no association between mercury and anxiety (34).

We used weighted quantile sum (WQS) regression to explore the effect of combined exposure to the three metals on anxiety and found that a positive WQS index was significantly associated with an increased risk of anxiety. In an analysis of urine metal-anxiety associations in American adults, a positive WQS index was significantly associated with anxiety risk (OR [95% CI]: 1.23 [1.04, 1.39]) (7). Another WQS analysis of the NHANES database examining blood heavy metal exposure and anxiety associations found that mixed metal exposure was positively associated with anxiety [*P* = 0.033, OR (95%): 1.437 (1.031, 2.003)] (33). These findings are consistent with our results.

Current studies have highlighted the positive effects of exercise on anxiety. In our study, the risk of anxiety following cadmium exposure was relatively lower in the vigorous/moderate RPA group compared to the light RPA group. Some studies suggest that physical activity is an effective way to address anxiety symptoms in children and adolescents (35). Research on the relationship between physical activity and anxiety indicates that low physical activity levels are associated with an increased prevalence of anxiety (36). Other studies have shown that physical activity can reduce the risk of anxiety (37, 38).

The mechanism of cadmium-induced anxiety remains unclear. Cadmium is a highly neurotoxic heavy metal that interferes with DNA repair mechanisms by generating reactive oxygen species (39). Animal experiments have shown that cadmium-treated mice have fewer adult cells, fewer adult neurons, and a reduced proportion of adult cells that differentiating into mature neurons in the dentate gyrus granules. This suggests that cadmium exposure from puberty to adulthood is sufficiently high to cause cognitive deficits and impair key processes of hippocampal neurogenesis in mice (18). Additionally, cadmium selenide quantum dot (CdSe QD) exposure may induce neurobehavioral toxicity and alter mRNA levels of dopamine and oxidative stress-related genes in developing animals, as demonstrated in a toxicological assessment of cadmium-containing quantum dots in developing zebrafish (40). Cadmium enters the nervous system and disrupts mitochondrial respiration by decreasing ATP synthesis and increasing the production of reactive oxygen species. It also impairs normal neurotransmission by increasing the asynchrony of neurotransmitter release and disrupting neurotransmitter signaling proteins, damages the blood-brain barrier and alters the regulation of glycogen metabolism (41). These neurotoxicities of cadmium may cause the onset of anxiety.

This study has several strengths. Firstly, the data come from three cycles of NHANES 2007–2012, a reliable data source, with a sufficiently large sample size. Secondly, the complex sampling with NHANES was considered in the statistical analyses, enhancing the credibility of the results. Finally, the use of standardized data collection and reliable information from the NHANES database increases the objectivity and reliability of the findings.

Our article has several limitations. Firstly, NHANES data are cross-sectional, which prevents an in-depth exploration of causality. Future cohort studies are necessary to confirm our conclusions. Secondly, although the confidence level of the dependent variable was high, the measurements were obtained from questionnaires and may be influenced by the subjectivity of respondents. Lastly, as the data were sourced from the United States, caution should be exercised when generalizing the findings to other populations.

## 5 Conclusion

High levels of blood cadmium are positively associated with the development of anxiety disorders, which needs to be further verified in future studies.

## Data availability statement

The datasets presented in this study can be found in online repositories. The names of the repository/repositories and accession number(s) can be found below: https://www.cdc.gov/nchs/nhanes/index.htm.

## Ethics statement

The studies involving humans were approved by National Center for Health Statistics Research Ethics Review Board. Written informed consent for participation was not required for this study in accordance with the national legislation and the institutional requirements. The studies were conducted in accordance with the local legislation and institutional requirements. Written informed consent for participation in this study was provided by the participants' legal guardians/next of kin. Written informed consent was obtained from the individual(s), and minor(s)' legal guardian/next of kin, for the publication of any potentially identifiable images or data included in this article.

## Author contributions

LB: Writing – original draft, Writing – review & editing. ZW: Writing – review & editing. YZ: Writing – review & editing. HJ: Validation, Writing – review & editing. JS: Writing – review & editing. JC: Supervision, Writing – review & editing.
