# Supplementary material for: Association of blood cadmium, lead, and mercury with anxiety: a cross-sectional study from NHANES 2007–2012
Source: Front Public Health. 2024 Aug 12;12:1402715. doi: 10.3389/fpubh.2024.1402715 (PMC11345141; doi:10.3389/fpubh.2024.1402715)
Supplement: Supplementary file 1 [file Table_1.DOCX]

**Supplementary materials**

**Table S1** Summary statistics for three metals

| Blood metal | Mean±SD | Q25-Q75 |
| --- | --- | --- |
| Cadmium(ug/L) | 0.54±0.61 | 0.22-0.62 |
| Lead(ug/dL) | 1.68±1.72 | 0.84-2.00 |
| Mercury(ug/L) | 1.55±2.44 | 0.47-1.71 |

Table S2 Bimetallic and polymetallic models for heavy metals, weighted

|  | Q1 | Q2 | Q3 | Q4 |
| --- | --- | --- | --- | --- |
| Cd+Pb |  |  |  |  |
| Cd | Ref | 1.048(0.919,1.195) | 0.939(0.819,1.078) | 1.286(1.115,1.483) ** |
| Pb | Ref | 1.083(0.938,1.251) | 0.959(0.802,1.147) | 0.973(0.843,1.123) |
| Cd+Hg |  |  |  |  |
| Cd | Ref | 1.045(0.916,1.193) | 0.938(0.819,1.074) | 1.280(1.112,1.472) ** |
| Hg | Ref | 0.954(0.819,1.112) | 1.026(0.886,1.188) | 1.027(0.882,1.196) |
| Pb+Hg |  |  |  |  |
| Pb | Ref | 1.099(0.954,1.267) | 0.982(0.826,1.168) | 1.017(0.882,1.172) |
| Hg | Ref | 0.944(0.812,1.098) | 1.009(0.870,1.170) | 1.008(0.863,1.177) |
| Cd+Pb+Hg |  |  |  |  |
| Cd | Ref | 1.047(0.919,1.193) | 0.939(0.818,1.078) | 1.288(1.117,1.484) ** |
| Pb | Ref | 1.079(0.932,1.249) | 0.956(0.801,1.141) | 0.968(0.838,1.117) |
| Hg | Ref | 0.954(0.818,1.113) | 1.022(0.879,1.188) | 1.028(0.883,1.198) |

**:p<0.05;**:p<0.01;***:p<0.001;*

*Cd: Cadmium; Pb: Lead; Hg: Mercury*

*Adjusted for gender, age, race, educational, marital status, smoking status, drinking status, body mass index, poverty income ratio,* *hypertension, diabetes, and physical activity.*

| WQS | positive weights | Negative weights |
| --- | --- | --- |
| OR (95%CI) | 1.086(1.016,1.160) * | 0.959(0.890,1.032) |
| Cadmium | 0.823 | 0.027 |
| Lead | 0.018 | 0.566 |
| Mercury | 0.158 | 0.408 |

Table S3 Heavy metal co-exposure effects and metal weights under the WQS model

**:p<0.05;**:p<0.01;***:p<0.001;*

*Adjusted for gender, age, race, educational, marital status, smoking status, drinking status, body mass index, poverty income ratio, hypertension, diabetes, and physical activity.*
